# HSV-2 Infection of Human Genital Epithelial Cells Upregulates TLR9 Expression Through the SP1/JNK Signaling Pathway

**DOI:** 10.3389/fimmu.2020.00356

**Published:** 2020-03-04

**Authors:** Kai Hu, Ming Fu, Jun Wang, Sukun Luo, Mariana Barreto, Rubin Singh, Tasnim Chowdhury, Mei Li, Mudan Zhang, Xinmeng Guan, Juhua Xiao, Qinxue Hu

**Affiliations:** ^1^Institute for Infection and Immunity, St George's, University of London, London, United Kingdom; ^2^State Key Laboratory of Virology, Wuhan Institute of Virology, Chinese Academy of Sciences, Wuhan, China; ^3^University of Chinese Academy of Sciences, Beijing, China; ^4^Institute for Clinical Research Center, Wuhan Medical and Healthcare Center for Women and Children, Wuhan, China; ^5^Department of Ultrasound, Jiangxi Provincial Maternal and Child Health Hospital, Nanchang, China

**Keywords:** herpes simplex virus type 2, toll-like receptor 9, genital epithelial cells, specificity protein 1, JNK pathway

## Abstract

It is known that herpes simplex virus type 2 (HSV-2) triggers the activation of Toll-like receptor (TLR) 9 signaling pathway and the consequent production of antiviral cytokines in dendritic cells. However, the impact of HSV-2 infection on TLR9 expression and signaling in genital epithelial cells, the primary HSV-2 targets, has yet to be determined. In the current study, by using both human genital epithelial cell lines and primary genital epithelial cells as models, we found that HSV-2 infection enhances TLR9 expression at both mRNA and protein levels. Such enhancement is virus replication-dependent and CpG-independent, while the HSV-2-mediated upregulation of TLR9 does not activate TLR9 signaling pathway. Mechanistically, a SP1 binding site on TLR9 promoter appears to be essential for HSV-2-induced TLR9 transactivation. Upon HSV-2 infection, SP1 translocates from the cytoplasm to the nucleus, and consequently binds to TLR9 promoter. By using specific inhibitors, the JNK signaling pathway is shown to be involved in the HSV-2-induced TLR9 transactivation, while HSV-2 infection increases the phosphorylation but not the total level of JNK. In agreement, antagonism of JNK signaling pathway inhibits the HSV-2-induced SP1 nuclear translocation. Taken together, our study demonstrates that HSV-2 infection of human genital epithelial cells promotes TLR9 expression through SP1/JNK signaling pathway. Findings in this study provide insights into HSV-2-host interactions and potential targets for immune intervention.

## Introduction

Herpes simplex virus type 2 (HSV-2) is a large double-stranded DNA (dsDNA) virus that primarily infects genital epithelial cells during the lytic cycle and can also eslishes a lifelong latency in the sacral ganglia (1). HSV-2 infection causes clinical manifestations such as genital ulcers, blindness and encephalitis (2, 3). HSV-2 is epidemiologically proven to enhance HIV-1 acquisition, transmission and disease progression (4–6). An implication of HSV-2 to the pathogenesis of Kaposi's sarcoma has also been proposed (7). To date, there are still no preventative vaccines or curative measures available against HSV-2.

During its infection and replication, HSV-2 can trigger innate immunity through various recognition signaling pathways including Toll-like receptor (TLR)-dependent and independent pathways (8–10). TLRs are type I transmembrane proteins that recognize pathogen-associated molecular patterns (PAMPs) and signal via MyD88-dependent or TRIF-dependent pathways. Different TLRs can recognize different HSV-2 components. For instance, TLR2 recognizes glycoproteins gH/gL and gB (11), TLR3 recognizes viral double-stranded RNA (dsRNA) (12) and TLR9 recognizes unmethylated CpG motifs in viral dsDNA (13). Activation of TLR-mediated signaling pathways leads to the production of inflammatory cytokines.

Recognition of HSV-2 by TLRs, in particular TLR9, has been reported by several studies. For instance, in plasmacytoid dendritic cells (pDCs), both HSV-1 and HSV-2 can stimulate IFN-α expression via TLR9/MyD88 signaling pathway, and this stimulation is not viral replication-dependent (13). Certain strains of HSV-1/2 are sequentially recognized by TLR2 and TLR9 in conventional DCs (cDCs), but not pDCs, for the induction of IL-6 and IL-12 (14). However, most of these studies adopted immunocompetent cells like DCs as models, and discrepancies have been observed (15). Given that HSV-2 predominantly infects epithelial cells at the portal of viral entry, the findings obtained from DCs may not well represent the events during its primary infection. In addition, previous studies mainly focused on whether and how HSV-2 infection triggers the activation of TLR9 signaling pathway, with little attention being paid to the regulation of TLR9 expression.

In this study, by using human genital epithelial cell lines and primary genital epithelial cells as models, we investigated the impact of HSV-2 infection on TLR9 expression and signaling. We demonstrated that HSV-2 upregulates TLR9 expression but does not activate TLR9 signaling pathway. We further revealed that HSV-2 enhances TLR9 expression in a viral replication-dependent manner by promoting TLR9 promoter activation via SP1/JNK signaling pathway.

## Materials and Methods

### Cells, Virus, and Inhibitors

Vero cell line and human cervical epithelial cell lines ME-180 and HeLa were purchased from American Type Culture Collection (ATCC) and cultured in DMEM supplemented with 10% FBS and antibiotics. HSV-2 (G strain) was obtained from LGC standards, propagated in ME-180, and titrated in Vero cells. Titrated virus stocks were aliquoted and stored at −80°C until use. Signaling inhibitors specifically targeting TBK1/IKKε (BX795), IκB-α (BAY11-7082), JNK (SP600125), and p38 (SB203580) were purchased from InvivoGen and used according to the manufacturer's instructions.

### Plasmids

Human TLR9 promoter sequence (−2,577/+77) was amplified from genomic DNA and cloned into pGL3-Basic luciferase reporter vector (Promega) and designated as pGL3-TLR9. Truncations on TLR9 promoter were made based on pGL3-TLR9, and designated as (−1,577/+77)TLR9, (−1,077/+77)TLR9, (−577/+77)TLR9, (−377/+77)TLR9, (−177/+77)TLR9, and (−77/+77)TLR9, respectively. Mutations of transcription factor binding sequences on TLR9 promoter were made based on full length pGL3-TLR9 using QuickChange II Site-Directed Mutagenesis Kit (Agilent), and designated as 5′PU MUT, 3′PU MUT, 3′AP MUT, 5′AP+3′AP MUT, SP1 MUT and C/EBP MUT, respectively. Full open reading frames of transcription factor specificity protein 1 (SP1) and TLR9 were amplified from human cDNA library and cloned into pcDNA3.1(+) (Thermo Fisher Scientific) and designated as pcDNA3.1-SP1 and pcDNA3.1-TLR9, respectively. The pRL-TK Renilla luciferase control reporter vector was purchased from Promega. All primers used for plasmid construction were listed in Table S1.

### Isolation and Infection of Primary Foreskin Epithelial Cells

All protocols involving human subjects were reviewed and approved by the Research Ethics Committee of Wuhan Institute of Virology, Chinese Academy of Sciences. Informed written consents from the human subjects were obtained in this study, and informed written parental consents were obtained for all participants under the age of 18.

Foreskin samples were obtained from teenagers who underwent circumcision in Wuhan Medical and Healthcare Center for Women and Children, and foreskin epithelial cells were isolated as previously described (16). For infection assay, cells were infected with HSV-2 at an MOI of 0.5 for 24 h. For signaling pathway inhibition, inhibitors were introduced into the culture 1–2 h after infection and maintained until 24 h. After 24 h of infection, cells were lysed and TLR9 expression was determined by Western blot.

### HSV-2 Infection

Human cervical epithelial cell line ME-180 or primary foreskin epithelial cells were preseeded in 24-well-plates 1 day before infection. In most cases, cells were infected with HSV-2 at an MOI of 0.5 for 24 h. For infection dose assay, cells were infected with ascendant HSV-2 doses ranging from 0 to 2 MOI. For infection time course, cells were infected with HSV-2 for ascendant time periods ranging from 0 to 30 h. UV-inactivated HSV-2 (UV-HSV-2) was obtained by exposing viruses to UV irradiation for 15 min as previously described (17). To separate HSV-2 virus particles from cytokine-containing medium, virus stocks were filtrated through a 100 kD ultracentrifugal filter tube (Thermo Fisher Scientific Pierce) by centrifuging at 1,000 g for 20 min at 4°C. Filter-through fraction (HSV-2 free cytokine fraction) was collected directly while the membrane-retained fraction (cytokine-free HSV-2 fraction) was diluted with fresh medium and collected for infection.

### Transfection and Luciferase Reporter Gene Assay

All plasmid transfections in this study were conducted using Lipofectamine 2000 (Thermo Fisher Scientific Invitrogen) according to the manufacturer's instructions. For luciferase reporter gene-based promoter activation, constructs carrying promoter of interest in full-length, truncations or with mutations were co-transfected with the Renilla-expressing control plasmid pRL-TK into ME-180 cells. Four to six hours post transfection, cells were infected with HSV-2 or UV-HSV-2 for another 24 h. Afterwards, cells were lysed and firefly luciferase and Renilla luciferase activities were measured using the Dual Luciferase Assay Kit (Promega) according to the manufacturer's instructions. For siRNA knockdown, siRNAs were transfected 24 h before plasmid transfection using X-tremeGENE™ siRNA Transfection Reagent (Roche) according to the manufacturer's instructions. For signaling pathway inhibition, signaling pathway inhibitors were added 1–2 h after virus infection and maintained until luciferase measurement. For CpG treatment, 4–6 h after plasmid transfection, cells were treated with CpG (ODN 2395, Miltenyi Biotec) or GpC (ODN 5328, Miltenyi Biotec) according to the manufacturer's instructions.

### RNA Extraction and Semi-Quantitative RT-PCR

Total RNA was extracted using RNeasy Mini Kit (Qiagen) and then reverse-transcribed into cDNA using High-capacity cDNA Reverse Transcription Kit (Thermo Fisher Scientific), both according to the manufacturers' instructions. TLR9 mRNA level was semi-quantified by SYBR Green RT-PCR, as previously described with modifications (18). In brief, reaction was prepared using SsoAdvanced™ Universal SYBR Green Supermix (Bio-Rad) and PCR was run on a Bio-Rad CFX Connect platform. GAPDH was used as an internal control and 2^−ΔΔCt^ was used to calculate the relative expression of TLR9. The following primer pairs were used. TLR9, forward: 5′-CGTCTTGAAGGCCTGGTGTTGA-3′, reverse: 5′-CTGGAAGGCCTTGGTTTTAGTGA-3′; GAPDH, forward: 5′-GCCAAGGTCATCCATGACAACTTTGG-3′, reverse: 5′-GCCTGCTTCACCACCTTCTTGATGTC-3′.

### ELISA

IL-6 expression by ME-180 and peripheral blood mono-nuclear cells (PBMCs) in response to HSV-2 infection or GpC stimulation was quantified by ELISA. In brief, PBMCs were isolated from single buffy coats of healthy donors obtained from NHS Blood and Transplant using Histopaque (Sigma-Aldrich). ME-180 cells with or without TLR9 overexpression and PBMCs were either infected with HSV-2 or stimulated with CpG or control GpC for 24 h. Cell culture supernatants were collected and IL-6 was quantified by ELISA using human IL-6 ELISA kit (Thermo Fisher Scientific) according to the manufacturer's instructions.

### Western Blot

For detection of protein expression at whole cell level, cell lysates were prepared using Pierce IP Lysis buffer (Thermo Fisher Scientific) supplemented with protease inhibitor cocktail (Roche) and phosphatase inhibitor cocktail (Santa Cruz). For detection of protein expression in cytoplasm and nucleus, cytoplasmic and nuclear fractions were prepared using Pierce NE-PER Nuclear and Cytoplasmic Extraction Kit (Thermo Fisher Scientific), according to the manufacturer's instructions. For Western blot analysis, protein samples were first separated by 4–15% SDS-PAGE gel (Bio-Rad), and then transferred onto a 0.45 μm PVDF membrane. After blocking with 5% non-fat milk, membrane was incubated sequentially with primary and HRP-conjugated secondary antibodies. Following the final incubation, membrane was extensively washed, and immuno-bands were visualized using ECL substrate (Millipore) under a CCD camera (LAS 4000, Fujifilm). The following primary antibodies were used: mouse anti-human TLR8 (Santa Cruz), rabbit anti-human TLR9 (Cell Signaling Technology), mouse anti-human β-actin (Santa Cruz), rabbit anti-human MyD88 (Cell Signaling Technology), mouse anti-human SP1 (Santa Cruz), mouse anti-human HDAC1 (Santa Cruz), mouse anti-human JNK (Santa Cruz), rabbit anti-human p-JNK (Cell Signaling Technology). The following HRP-conjugated secondary antibodies were used: goat anti-mouse IgG-HRP and goat anti-rabbit IgG-HRP (both from Abcam).

### Chromatin Immunoprecipitation (ChIP) Assay

ChIP was used to test the binding of SP1 to TLR9 promoter using Piece Magnetic ChIP Kit (Thermo Fisher Scientific), as previously described with modifications (19). In brief, cells with or without HSV-2 infection were crosslinked with 1% formaldehyde and harvested. Pelleted cells were then lysed and digested with MNase in the presence of protease/phosphatase inhibitors. After digestion, nuclear fraction was harvested, and fragmentized chromatin was released from nuclei by sonication. Immunoprecipitation was performed with a ChIP grade rabbit anti-SP1 antibody (Merck Millipore) or a normal rabbit IgG (negative control, Merck Millipore) overnight at 4°C and pulled down with protein A/G magnetic beads. Recovered DNA samples were used for PCR using TLR9 promoter specific primers. Forward: 5′-AAGAGGAAGGGGTGAAGGAG-3′, reverse: 5′-TTCCCACAGGGGCAGCAGCG-3′.

### Statistical Analysis

All data in this study were expressed as mean ± standard deviation (SD). Statistical analyses were performed with GraphPad Prism 7.02. Mann–Whitney test was used for comparison between two groups while Kruskal–Wallis test was used when three or more groups were compared. For all comparisons, a *p* < 0.05 was considered statistically significant.

## Results

### HSV-2 Infection Increases TLR9 Transcription and Translation

**Figure 1:**
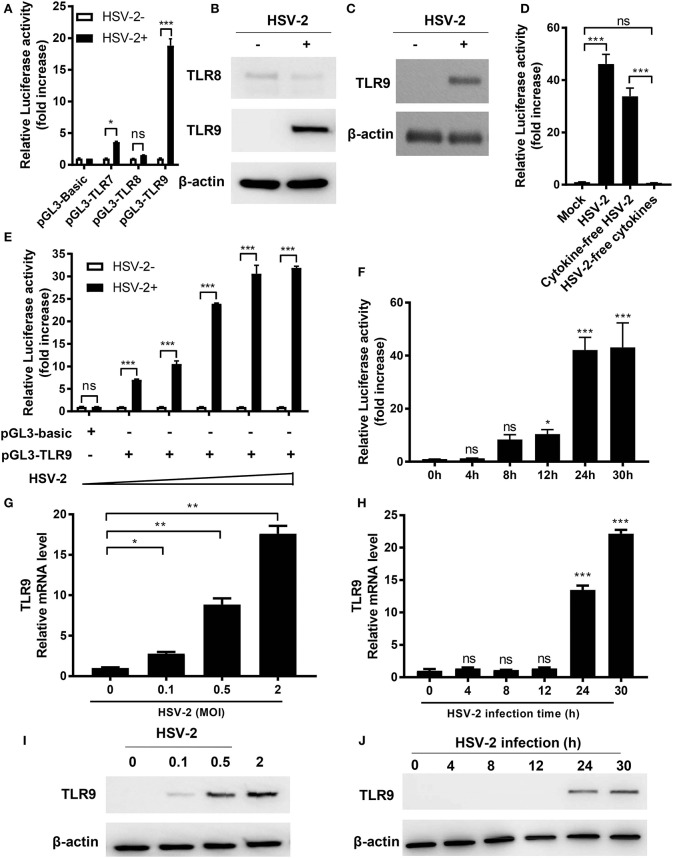
HSV-2 infection induces TLR9 expression in genital epithelial cells. **(A)** ME-180 cells were transfected with reporter plasmid pGL3-TLR7, pGL3-TLR8 or pGL3-TLR9 and infected with or without HSV-2. Twenty-four hours later, relative luciferase activity was measured. Data shown are mean ± SD of three independent experiments with each condition performed in duplicate. **(B,C)** ME-180 **(B)** and primary foreskin epithelial cells **(C)** were infected with HSV-2 for 24 h and the expression of TLR8 and TLR9 was determined by Western blot. One representative experiment out of three is shown. **(D)** HSV-2 stock was fractionized into cytokine-free viruses and virus-free cytokines by ultrcentrifugation and both fractions were used to infect ME-180 cells transfected with pGL3-TLR9. Twenty-four hours after infection, relative luciferase activity was measured. Data shown are mean ± SD of three independent experiments with each condition performed in duplicate. **(E,F)** ME-180 cells were transfected with or without pGL3-TLR9 were infected with ascending doses of HSV-2 for 24 h **(E)** or with 0.5 MOI HSV-2 for ascending infection time periods **(F)**. After incubation, relative luciferase activity was measured. Data shown are mean ± SD of three independent experiments with each condition performed in duplicate. **(G–J)** ME-180 cells were infected with or without ascending doses of HSV-2 for 24 h **(G,I)** or infected with 0.5 MOI HSV-2 for ascending infection time periods **(H,J)**. After incubation, TLR9 mRNA level **(G,H)** and protein level **(I,J)** were determined by RT-PCR **(G,H)** and Western blot **(I,J)**, respectively. For RT-PCR results, data shown are mean ± SD of three independent experiments with each condition performed in duplicate. For Western blot results, one representative experiment out of three is shown. ns, statistically not significant; ^*^p < 0.05; ^**^p < 0.01; ^***^p < 0.001.

It is known that HSV-2 activates several TLRs in pDCs (13). Here we investigated the impact of HSV-2 infection on TLR7, 8, and 9 activation in human genital epithelial cells, the main HSV-2 targets during primary infection. We constructed luciferase-carrying plasmids under the control of TLR7, 8 or 9 promoter (named as pGL3-TLR7, pGL3-TLR8, and pGL3-TLR9, respectively) and examined the responses to HSV-2 infection in cervical epithelial cells ME-180. As shown in Figure 1A, HSV-2 infection significantly induced TLR9 promoter activation. After HSV-2 infection, TLR7 promoter was also moderately activated but no apparent activation was observed for TLR8 promoter. Since TLR9 promoter showed the highest level of activation upon HSV-2 infection, we focused on HSV-2 infection-induced TLR9 upregulation. Western blot results showed that HSV-2-induced activation of TLR9 promoter also led to the increase of TLR9 expression at protein level in both ME-180 (Figure 1B) and primary foreskin epithelial cells (Figure 1C).

To exclude possible involvement of cytokines in the virus stock, HSV-2 virus stock was filtered through a 100 kD Amicon ultracentrifugal unit. Cytokine-free viruses and virus-free supernatants were harvested separately and used to treat cells transfected with pGL3-TLR9. Results showed that only virus-containing fraction (cytokine-free HSV-2), but not HSV-2-free cytokines induced TLR9 promoter activation, indicating that the TLR9 induction was mediated by HSV-2 but not cytokines in the samples (Figure 1D).

Further infection dose assay showed that TLR9 promoter activation was enhanced when HSV-2 dose increased (Figure 1E). Time-course assay revealed that HSV-2 induced TLR9 promoter activation in an infection time-dependent manner, which peaked around 24 h after infection (Figure 1F). Consistent results were also observed at both mRNA and protein levels (Figures 1G–J). In addition, ME-180 cells infected with 0.5 MOI of HSV-2 showed a much higher percentage of infection compared to those infected with 0.1 MOI of HSV-2, which was consistent with the levels of TLR9 mRNA and protein, suggesting that TLR9 expression was upregulated in HSV-2-infected cells (Figures 1G,I and Figure S1).

Taken together, our data indicate that HSV-2 induces the activation of TLR9 promoter and consequently leads to the upregulation of TLR9 at both the mRNA and protein levels.

### HSV-2-Induced TLR9 Upregulation Is Viral Replication-Dependent

**Figure 2:**
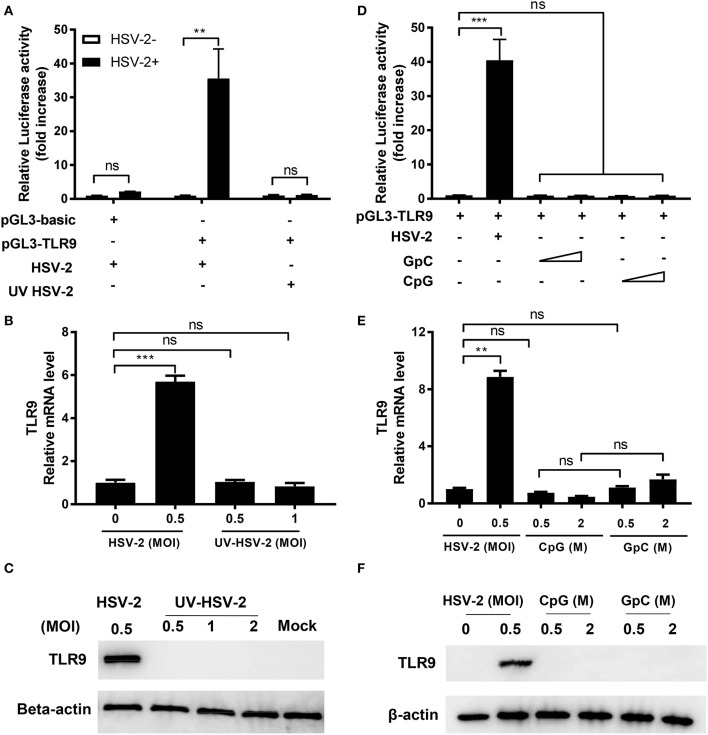
HSV-2-induced TLR9 expression is viral replication-dependent. ME-180 cells were transfected with or without pGL3-TLR9 and infected with HSV-2 **(A–C)** or treated with CpG or GpC ODNs **(D–F)**. Twenty-four hours after infection or treatment, relative luciferase activity **(A,D)**, relative TLR9 mRNA level **(B,E)**, and TLR9 protein level **(C–F)** were determined by dual luciferase activity assay **(A,D)**, RT-PCR **(B,E)**, and Western blot **(C,F)**, respectively. For luciferase assay and RT-PCR, data shown are mean ± SD of three independent experiments with each condition performed in duplicate. For Western blot results, one representative experiment out of three is shown. ns, statistically not significant; ^**^p < 0.01; ^***^p < 0.001.

As a pattern recognition receptor, TLR9 is activated by unmethylated CpG-rich DNA sequence and leads to the secretion of pro-inflammatory cytokines. HSV-2, as a DNA virus, has abundant CpG motifs in its genome. To assess if HSV-2-induced TLR9 upregulation was a result of CpG-triggered TLR9 activation, TLR9 induction by UV inactivated HSV-2 and synthesized CpG ODNs was analyzed. As shown in Figures 2A,D, only replicative HSV-2, but not UV-treated HSV-2 or CpG ODN triggered the activation of TLR9 promoter. Consistent results were observed at both mRNA and protein levels (Figures 2B,C,E,F). These data indicate that HSV-2-induced TLR9 upregulation is HSV-2 replication-dependent and CpG-independent.

### HSV-2 Induces TLR9 Expression Without Activating TLR9 Signaling Pathway

**Figure 3:**
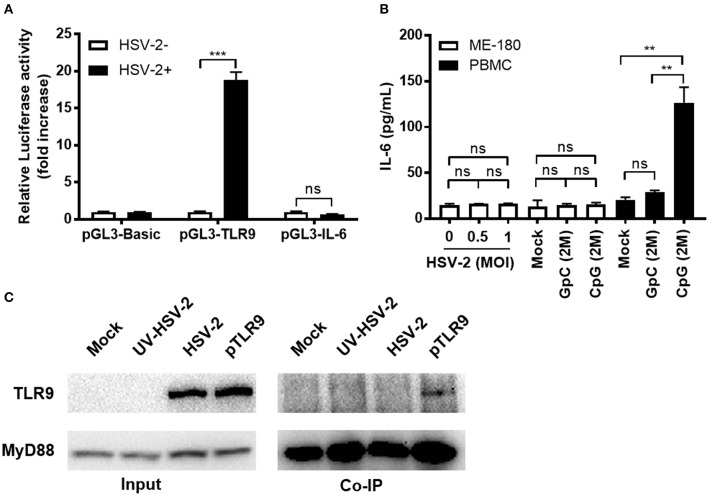
HSV-2-induced TLR9 expression does not activate TLR9 signaling pathway. **(A)** ME-180 cells were transfected with pGL3-TLR9 or pGL3-IL-6 and infected with 0.5 MOI HSV-2 for 24 h. After incubation, relative luciferase activity was measured. Data shown are mean ± SD of three independent experiments with each condition performed in duplicate. **(B)** ME-180 cells or PBMCs were infected with or without HSV-2 or treated with or without CpG or GpC ODNs for 24 h. After incubation, IL-6 concentration in the cell culture supernatants were measured by ELISA. Data shown are mean ± SD of three independent experiments with each condition performed in duplicate. ns, statistically not significant; ^**^p < 0.01; ^***^p < 0.001. **(C)** ME-180 cells were either mock-infected, or infected with UV-HSV-2, HSV-2 or transfected with pTLR9. Twenty-four hours later, cells were lysed and immunoprecipitation was performed with anti-MyD88 antibody and the presence of TLR9 and MyD88 in the pulldown was detected by Western blot. One representative experiment out of three is shown.

Since HSV-2 infection induced TLR9 expression, we next investigated whether this induction could activate TLR9 signaling pathway and cause the production of pro-inflammatory cytokines like IL-6. We constructed a firefly luciferase reporter plasmid under the control of IL-6 promoter and tested its response to HSV-2 infection. Our data showed that HSV-2 infection induced the promoter activation of TLR9 but not that of IL-6 (Figure 3A). To further confirm these results, we treated ME-180 cells with HSV-2 or CpG ODNs and measured the IL-6 concentration in the supernatants. As shown in Figure 3B, neither HSV-2 infection nor CpG treatment induced IL-6 expression. As a positive control, CpG treatment significantly increased IL-6 in the PBMCs (Figure 3B). Upon activation, TLR9 binds to its adaptor protein MyD88, which subsequently activates downstream signaling pathway. To assess if HSV-2 infection-upregulated TLR9 was activated and able to bind to MyD88, co-immunoprecipitation with anti-MyD88 antibody was performed. As shown in Figure 3C, no interaction between HSV-2-upregulated TLR9 and MyD88 was detected. However, TLR9 overexpression by transfection with pTLR9 plasmid showed binding of the two proteins. In consistent, pTLR9 transfection alone also triggered high level of IL-6 expression (Figure S2). Taken together, these data indicate that HSV-2 infection induces TLR9 expression without activating the TLR9 signaling pathway.

### SP1-Binding Site in TLR9 Promoter Is Involved in HSV-2-Induced TLR9 Expression

**Figure 4:**
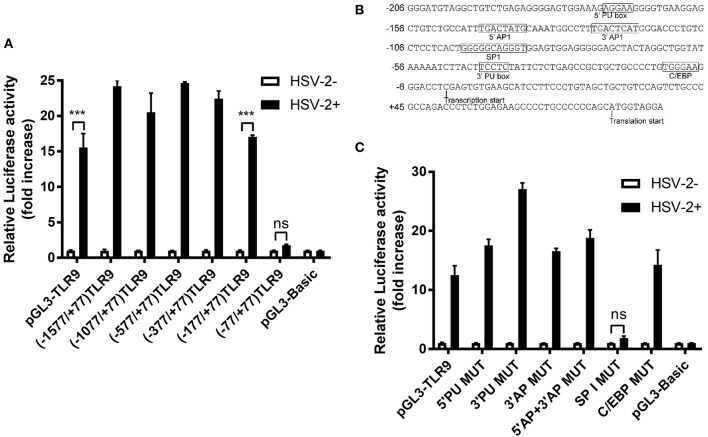
SP1 binding site on TLR9 promoter is involved in HSV-2-induced TLR9 transactivation. **(A)** ME-180 cells were transfected with reporter plasmids with 5′ serial deletions within the TLR9 promoter region and infected with or without 0.5 MOI HSV-2 for 24 h. After incubation, relative luciferase activity was measured. Data shown are mean ± SD of three independent experiments with each condition performed in duplicate. **(B)** TLR9 promoter sequence was analyzed by Gene2promoter software and the predicted transcription binding sites were shown. **(C)** ME-180 cells were transfected with reporter plasmids with or without mutations within the TLR9 promoter region and infected with or without 0.5 MOI HSV-2 for 24 h. After incubation, relative luciferase activity was measured. Data shown are mean ± SD of three independent experiments with each condition performed in duplicate. ns, statistically not significant; ^***^p < 0.001.

Since TLR9 promoter could be transactivated by HSV-2 infection, we next investigated whether one or more *cis*-elements in the TLR9 promoter was involved in this transactivation. 5' serial truncations of TLR9 promoter showed that HSV-2 induced luciferase activity was lost when−177 to−77 bp of the promoter were deleted (Figure 4A). Additional bioinformatics analysis predicted a few transcription factor binding sites in this region, including PU box, AP1, SP1, and C/EBP (Figure 4B). Removal of these sites by point-direct mutations showed that only the mutation of the SP1 binding site demolished the responsiveness of TLR9 promoter to HSV-2 infection, indicating that the SP1 binding site within−177 to−77 bp is essential for the HSV-2-induced TLR9 promoter activation (Figure 4C).

### SP1 Binds to TLR9 Promoter After HSV-2 Infection

**Figure 5:**
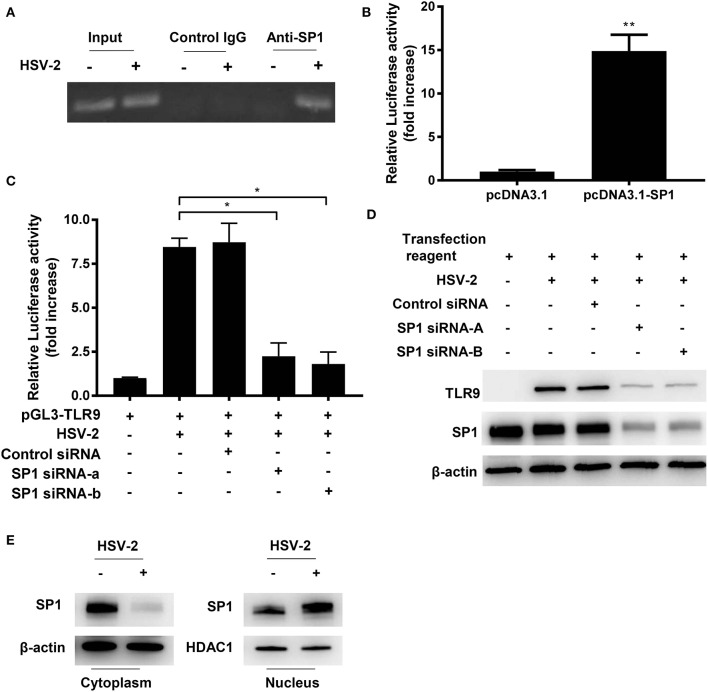
HSV-2 infection promotes SP1 phosphorylation and nuclear translocation. **(A)** ME-180 cells were infected with or without 0.5 MOI HSV-2 for 24 h and chromatin immunoprecipitation assay was performed using anti-SP1 antibody and control IgG. The amplification of input samples was also shown. One representative experiment out of three is shown. **(B)** ME-180 cells co-transfected with pGL3-TLR9 and pcDNA3.1-SP1 or pcDNA3.1 for 24 h, and then relative luciferase activity was measured. Data shown are mean ± SD of three independent experiments with each condition performed in duplicate. **(C,D)** ME-180 cells were sequentially transfected with SP1 siRNAs or control siRNA **(C,D)** and pGL3-TLR9 **(C)**, and then infected with HSV-2. Twenty-four hours after infection, relative luciferase activity **(C)** and TLR9 and SP1 expression were determined by dual luciferase assay **(C)** and Western blot **(D)**, respectively. For luciferase assay and RT-PCR, data shown are mean ± SD of three independent experiments with each condition performed in duplicate. For Western blot results, one representative experiment out of three is shown. **(E)** ME-180 cells were infected with or without HSV-2 for 24 h. Cell cytoplasmic and nuclear fractions were isolated, and SP1 expression was determined by Western blot. One representative experiment out of three is shown. ns, statistically not significant; ^*^p < 0.05; ^**^p < 0.01.

Given the importance of SP1 binding site in TLR9 transactivation upon HSV-2 infection, we proposed that HSV-2 infection likely activates TLR9 promoter by promoting SP1 binding to SP1 binding site in the promoter. To test this hypothesis, ChIP assay was performed and TLR9 promoter fragment containing the SP1 binding site was amplified by PCR. As shown in Figure 5A, a positive amplification of the fragment was only seen in the pulldown by anti-SP1 antibody in HSV-2 treated cells, but not by control IgG or in cells without HSV-2 treatment. This indicates that HSV-2 infection enhances TLR9 expression through promoting SP1 binding to TLR9 promoter.

To further confirm the role of SP1 on TLR9 promoter transactivation, the activation level of TLR9 promoter was measured under the condition of SP1 overexpression. As shown in Figure 5B, SP1 overexpression significantly enhanced TLR9 promoter activity. The essential role of SP1 in HSV-2-induced TLR9 transactivation was also confirmed by siRNA interference of SP1. Luciferase reporter gene assay showed that HSV-2-induced transactivation of TLR9 promoter was largely impaired when SP1 was knocked down by siRNAs (Figure 5C). Western blot analysis showed consistent results, revealing a SP1-dependent TLR9 expression under the condition of HSV-2 infection (Figure 5D). In order to regulate promoter activity, transcription factors need to translocate from the cytoplasm to the nucleus. We next investigated whether HSV-2 infection also enhanced SP1 nuclear translocation. As shown in Figure 5E, a near full nuclear translocation of SP1 was observed when cells were infected with HSV-2. In addition, upon HSV-2 infection, an increased SP1 phosphorylation level (top band) (20) was seen in the nuclear fraction, indicating that HSV-2 infection also enhanced SP1 phosphorylation (Figure 5E). Taken together, these data here indicate that HSV-2 infection induces TLR9 expression via promoting SP1 binding to TLR9 promoter.

### HSV-2 Induces TLR9 Expression by Activating JNK Signaling Pathway

**Figure 6:**
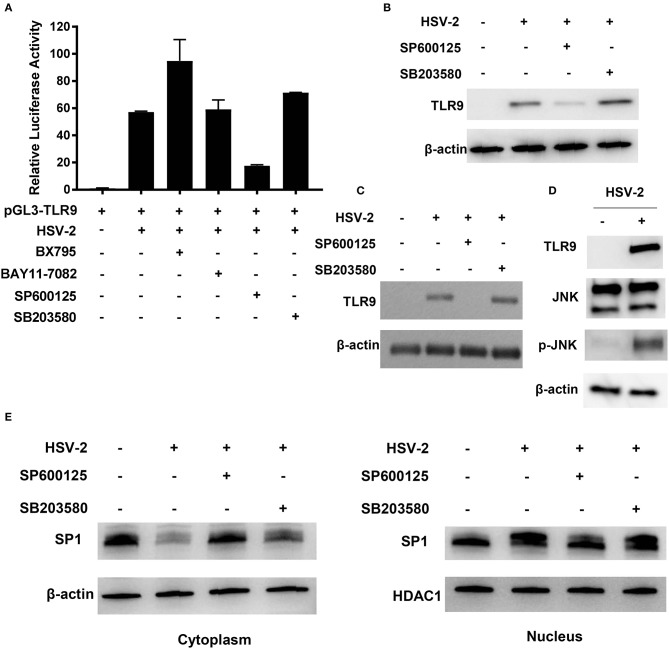
JNK signaling pathway is involved in HSV-2-induced TLR9 expression. **(A)** ME-180 cells were transfected with pGL3-TLR9, infected with HSV-2 and treated with various signaling pathway inhibitors. Twenty-four hours after treatment, relative luciferase activity was measured. Data shown are mean ± SD of three independent experiments with each condition performed in duplicate. **(B–E)** ME-180 **(B,D,E)** and primary foreskin epithelial cells **(C)** were infected with HSV-2 and treated with various signaling pathway inhibitors. Twenty-four hours later, TLR9 **(B,C)** and JNK and p-JNK **(D)** expression were determined by Western blot. **(E)** Cytoplasmic and nuclear fractions were isolated and SP1 expression was determined by Western blot. One representative experiment out of three is shown.

To determine which signaling pathway(s) was involved in HSV-2-induced TLR9 upregulation, inhibitors targeting TBK1/IKKε (BX795), IκBα (BAY11-7082), JNK (SP600125), and p38/MAPK (SB203580) were used. As shown in Figure 6A, only the addition of SP600125 significantly reduced the luciferase activity, indicating a JNK-dependent signaling pathway. To further confirm these findings, signaling inhibition assay was repeated in both ME-180 and primary foreskin epithelial cells where TLR9 expression was determined by Western blot. In consistent with the luciferase reporter gene assay, SP600125, but not SB203580 could significantly decrease TLR9 expression in both ME-180 (Figure 6B) and foreskin epithelial cells (Figure 6C).

We next determined the impact of HSV-2 infection on the activation of JNK signaling pathway. As shown in Figure 6D, HSV-2 infection did not show apparent impact on JNK expression, but significantly increased its phosphorylation level. The impact of inhibiting JNK signaling pathway on SP1 phosphorylation and nuclear translocation was also investigated. As shown in Figure 6E, SP600125, but not SB203580, significantly decreased SP1 nuclear translocation. Furthermore, upon SP600125 treatment, SP1 phosphorylation level was also considerably decreased in the nucleus.

Collectively, our study has revealed that replicative, but not UV-inactivated HSV-2 induces TLR9 expression in human genital epithelial cells, and this induction is through promoting SP1 binding to TLR9 promoter via JNK signaling pathway.

## Discussion

It is known that HSV-2 is recognized by TLR2/3/9 signaling pathway in immunocompetent cells like DCs, NK cells and macrophages, leading to the production of antiviral inflammatory cytokines, such as type I IFNs, IL-6, and IL-12. However, little attention has been paid to the interaction between HSV-2 and TLRs in genital epithelial cells. In the current study, we reveal that HSV-2 infection triggers TLR9 expression in both human genital epithelial cell lines and primary cells. We demonstrate that HSV-2-induced TLR9 expression is mediated by promoting SP1 binding to TLR9 promoter via JNK signaling pathway. Since TLR9 expression is mainly manifested at 24 h post infection, it is possible that HSV-2 drives a response which subsequently activates TLR9 promoter. Although it remains to be determined whether a secondary response is involved in the HSV-2-induced TLR9 expression, we can largely rule out the involvement of HSV-2-induced cytokines in the process, as we found that cytokines in the viral stock did not activate TLR9 promoter. Given that HSV-2 in our current study was propagated in ME-180, the cell line also used for the subsequent infection experiments, the cytokine composition induced in the infection experiments would be similar to that in the viral stock and unlikely to impact TLR9 expression.

TLRs recognize pathogens and activate downstream signaling members to initiate innate immune responses. In general, increased TLR expression can increase pathogen recognition, which leads to enhanced immune response (21). For instance, upregulation of TLR4 expression by IL-27 enhances proinflammatory cytokine production in human monocytes (22). GM-CSF-promoted expression of TLR3 and TLR7 increases the release of IL-13 and IL-6 in mast cells (23). Of interest, in our current study, although a significant increase of TLR9 expression was observed when epithelial cells were infected with HSV-2, we did not observe an apparently increased activation of TLR9 signaling pathway. In contrast, TLR9 overexpression in the absence of HSV-2 did trigger the activation of the signaling pathway in genital epithelial cells, showing that TLR9 overexpression in ME-180 cells activated the TLR9 signaling, which resulted in enhanced IL-6 secretion in the cell culture (Figure S2). Moreover, we found that genital epithelial cells with ectopic overexpression of TLR9 were resistant to HSV-2 infection (Figure S3), indicating a TLR9 mediated anti-HSV-2 capability in the genital epithelial cells. Nevertheless, HSV-2-induced TLR9 expression appeared not to trigger the activation of the downstream signaling pathway, suggesting that HSV-2 may have evolved a mechanism to antagonize the TLR9 signaling pathway.

We found that HSV-2-induced TLR9 expression is HSV-2 replication-dependent and CpG-independent, indicating that TLR9 expression is induced by HSV-2 infection rather than the CpG motifs within the viral genome. It has been shown that TLR9 can antagonize affinity maturation by preventing B cells from antigen capture and presentation (24). TLR9 was also reported to negatively modulate antifungal functions in macrophages (25). Although further investigation is required, it is probable that HSV-2 increases TLR9 expression as a mechanism to interrupt host adaptive immune response. It is known that HSV-2 progeny virus packaging happens in the Golgi apparatus which uses intracellular membrane system for virus release (26). During this process, the virus usually adopts cellular functions and pathways to facilitate its release by specifically interacting with host cell lipids and proteins (27). Since TLR9 is mainly present in the intracellular vesicles and circulates within the membrane system (28, 29), future study is warranted to determine whether HSV-2 can hijack TLR9 to enhance virus transportation.

The impact of HSV-2 infection on TLR9 activation in pDCs has been previously described, the findings of which are quite different from those observed in our current study using genital epithelial cells as models. In pDCs, both live and UV-inactivated HSV-2 induced IFN-α production by activating TLR9/MyD88 signaling pathway (13), whereas in genital epithelial cells, only live HSV-2 upregulated TLR9 expression and neither live nor UV-inactivated HSV-2 activated TLR9 signaling pathway. Such discrepancy may be attributed to differences in cell targets. DCs as immunocompetent antigen-presenting cells have high level of TLR9 expression (30), and upon HSV-2 infection, TLR9 can be activated by viral dsDNA before viral antagonism takes place. In contrast to that in immune cells, TLR9 expression in epithelial cells is less ubiquitous (30). We observed that, prior to HSV-2 infection, TLR9 expression was hardly detectable in genital epithelial cells at both mRNA and protein levels. The lack of TLR9 expression may explain why UV-inactivated HSV-2 or CpG ODN was unable to activate TLR9 signaling pathway in genital epithelial cells.

It is not yet clear whether HSV-2 infection upregulated TLR9 expression is important to viral replication or simply a byproduct during its replication. However, the upregulated TLR9 may shed light on the understanding of HSV-2 enhanced HIV-1 infection. It has been reported that TLR9 can transactivate HIV-1 LTR and initiate viral replication (31, 32). In addition, TLR9 polymorphism is related to HIV-1 progression (33, 34). In a separate study, we have also observed that replicative HSV-2 but not UV-treated HSV-2 activates HIV-1 LTR and this activation is sensitive to TLR9 siRNA treatment (unpublished data). Although further investigations are needed, these findings imply that HSV-2 may promote HIV-1 replication in a TLR9-dependent manner.

In conclusion, our study has revealed that replicative, but not UV-inactivated HSV-2 induces TLR9 expression in genital epithelial cells, and that such induction is through promoting SP1 binding to TLR9 promoter via JNK signaling pathway.

## Data Availability Statement

The raw data supporting the conclusions of this article will be made available by the authors, without undue reservation, to any qualified researcher.

## Ethics Statement

The studies involving human participants were reviewed and approved by the Research Ethics Committee of Wuhan Institute of Virology, Chinese Academy of Sciences. Written informed consent to participate in this study was provided by the participants' legal guardian/next of kin.

## Author Contributions

KH and QH conceived the study and wrote the manuscript. KH, MF, JW, SL, MB, RS, TC, ML, MZ, XG, and JX conducted experiments and analyzed the data. All authors read and approved the manuscript.

### Conflict of Interest

The authors declare that the research was conducted in the absence of any commercial or financial relationships that could be construed as a potential conflict of interest.
