# Supplementary material for: HSV-2 Infection of Human Genital Epithelial Cells Upregulates TLR9 Expression Through the SP1/JNK Signaling Pathway
Source: Front Immunol. 2020 Mar 4;11:356. doi: 10.3389/fimmu.2020.00356 (PMC7065266; doi:10.3389/fimmu.2020.00356)
Supplement: Supplementary file 1 [file Table_1.DOCX]

Supplementary Material

## Supplementary Tables

Table S1. Primers used for plasmid construction

| **Primer** | **Sequence (5'🡪3')** | **Plasmid** |
| --- | --- | --- |
| IL-6 pro-KpnI-F | GA**GGTACC**CTTTCCCAGGCTAGGATAAC | pGL3-IL-6 |
| IL-6 pro-HindIII-R | AT**AAGCTT**AGCTGGGCTCCTGGAGGGGA |  |
| TLR7 pro-KpnI-F | AT**GGTACC**CCAGTCAGACCCAACACCTC | pGL3-TLR7 |
| TLR7 pro-XhoI-R | CG**CTCGAG**CTTTCTTGATGGCATGGAG |  |
| TLR8 pro-SacI-F | CG**GAGCTC**CATATATACATGATGATACA | pGL3-TLR8 |
| TLR8 pro-XhoI-R | CG**CTCGAG**GTTTCTGTTGTTCTAATTTT |  |
| TLR9 pro-KpnI-F | TA**GGTACC**CTTGGCTTGGGGCCCCTTCC | pGL3-TLR9 |
| TLR9 pro-HindIII-R | AT**AAGCTT**GCTGGGGGGCAGGGGCTTCT |  |
| TLR9 pro-1-KpnI-F | TA**GGTACC**CCAGCCAGATGCCTGGCACT | (-1577/+77)TLR9 |
| TLR9 pro-HindIII-R | As above |  |
| TLR9 pro-2-KpnI-F | TA**GGTACC**TGGGCCTGGAGAGCACTCAG | (-1077/+77)TLR9 |
| TLR9 pro-HindIII-R | As above |  |
| TLR9 pro-3-KpnI-F | TA**GGTACC**GGCCCTCCACGCATGAGGCC | (-577/+77)TLR9 |
| TLR9 pro-HindIII-R | As above |  |
| TLR9 pro-4-KpnI-F | TA**GGTACC**TGCAGGAGCCAAGACCTGAGGGTGG | (-377/+77)TLR9 |
| TLR9 pro-HindIII-R | As above |  |
| TLR9 pro-5-KpnI-F | TA**GGTACC**AAAGAGGAAGGGGTGAAGGAGCTGT | (-177/+77)TLR9 |
| TLR9 pro-HindIII-R | As above |  |
| TLR9 pro-6-KpnI-F | TA**GGTACC**GGGGAGCTACTAGGCTGGTATAAAA | (-77/+77)TLR9 |
| TLR9 pro-HindIII-R | As above |  |
| M5PU-F | ggcagacagctccttcaccccg**ctagc**tttccactcccctctcagac | 5’PU MUT |
| M5PU-R | gtctgagaggggagtggaaa**gctag**cggggtgaaggagctgtctgcc |  |
| M3PU-F | gggcagcagcggctcagagaataactagagtaagatttttataccagc**ctagt**agctc | 3’PU MUT |
| M3PU-R | gagct**actag**gctggtataaaaatcttactctagttattctctgagccgctgctgccc |  |
| M3AP-F | gtctgccatttgactatgcaaatggcctt**ctga**tcatgggaccctgtcct | 3’AP MUT |
| M3AP-R | aggacagggtcccatga**tcag**aaggccatttgcatagtcaaatggcagac |  |
| M53AP-F | aaggggtgaaggagctgtctgccatt**ctga**tatgcaaatggccttctgat | 5’AP+3’AP MUT |
| M53AP-R | atcagaaggccatttgcata**tcag**aatggcagacagctccttcacccctt |  |
| MSP1-F | cctgtcctcctcactgg**cata**agggtggagtggagggg | SP1 MUT |
| MSP1-R | cccctccactccaccct**tatg**ccagtgaggaggacagg |  |
| MCEBP-F | tgagccgctgctgcccctg**gatac**agggacctcgagtgtgaag | C/EBP MUT |
| MCEBP-R | cttcacactcgaggtccct**gtatc**caggggcagcagcggctca |  |
| SP1-EcoRI-F | GC**GAATTC**ATGAGCGACCAAGATCACTC | pcDNA3.1-SP1 |
| SP1-XhoI-R | AT**CTCGAG**TCAGAAGCCATTGCCACTGA |  |
| TLR9-BamHI-F | TTA**GGATCC**ATGGGTTTCTGCCGCAGCGCCCTGC | pcDNA3.1-TLR9 |
| TLR9-XbaI-R | TCG**TCTAGA**CTATTCGGCCGTGGGTCCCTGGCAG |  |

Note:

F, forward primer; R, reverse primer; Bold bases represent enzyme restriction sites or mutated sequences.

## Supplementary Figures


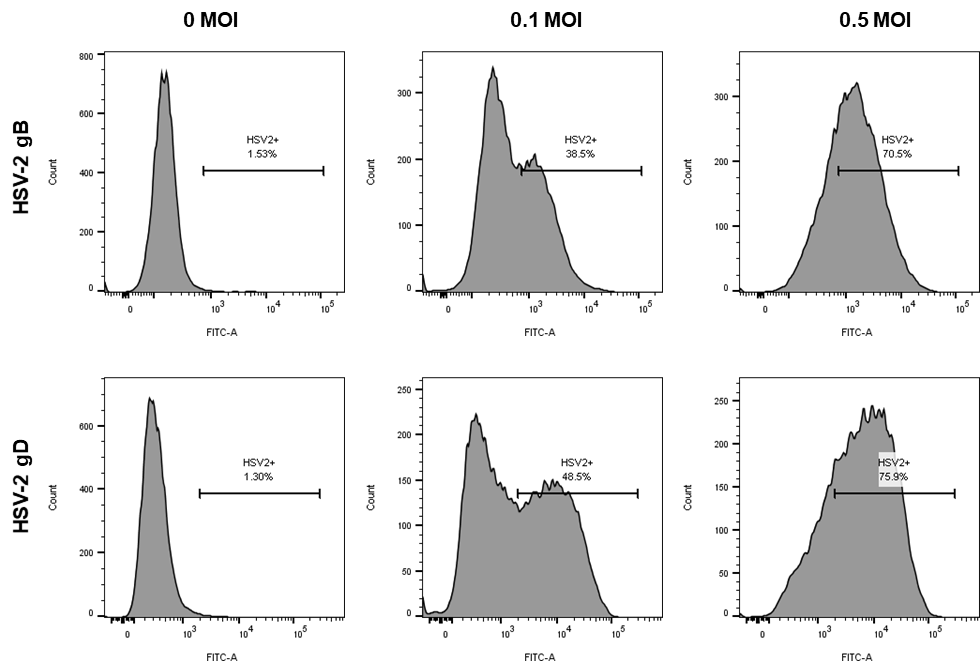


**Supplementary Figure 1.** Percentage of HSV-2 infected cells. ME-180 cells were either mock-infected, or infected with 0.1 or 0.5 MOI of HSV-2. Twenty-four hours later, the percentage of infected cells was assessed by flow cytometry with anti-gB and anti-gD antibodies, respectively, to determine HSV-2 gB/gD expression on cell surface. One representative experiment out of three is shown.

**Supplementary Figure 2.** Exogenous TLR9 expression activates TLR9 signaling pathway in genital epithelial cells. ME-180 cells were transfected with or without pcDNA3.1-TLR9 for 24 h and then treated with CpG or GpC ODNs for another 24 h. After incubation, IL-6 concentration in cell culture was measured by ELISA. Data shown are mean ± SD of three independent experiments with each condition performed in duplicate.

**Supplementary Figure 3.** Exogenous TLR9 expression inhibits HSV-2 entry in genital epithelial cells. ME-180 cells transfected with pcDNA3.1-TLR9 or control vector pcDNA3.1 for 24 h, and then infected with HSV-2. Forty-eight hours after infection, viral infection was determined by plaque assay. HSV-2 infection in the control group was considered as 100% infection. Data shown are mean ± SD of three independent experiments with each condition performed in duplicate.
